# Liver Fibrosis Regression and Associated Factors in HCV Patients Treated with Direct-Acting Antiviral Agents

**DOI:** 10.3390/life13091872

**Published:** 2023-09-05

**Authors:** Naim Abu-Freha, Osama Abu-Kosh, David Yardeni, Yaffa Ashur, Muhammad Abu-Arar, Baha Yousef, Shulamit Monitin, Sarah Weissmann, Ohad Etzion

**Affiliations:** 1 Institute of Gastroenterology and Hepatology, Soroka University Medical Center and the Faculty of Health Sciences, Ben-Gurion University of the Negev, Beer-Sheva 84101, Israel; yardeda@gmail.com (D.Y.); bahayo@clalit.org.il (B.Y.); shulamitmon@clalit.org.il (S.M.); ohadet34@yahoo.com (O.E.); 2 Division of Internal Medicine, Soroka University Medical Center, Beer-Sheva 84101, Israelabumuh@clalit.org.il (M.A.-A.); 3 Medical Management Unit, Soroka University Medical Center, Beer-Sheva 84101, Israel; 4 Soroka Clinical Research Center, Soroka University Medical Center, Beer-Sheva 84101, Israel; grzebins@post.bgu.ac.il

**Keywords:** hepatitis C, fibrosis, regression, direct-acting antiviral, Fibroscan

## Abstract

There is accumulating evidence that treatment of chronic hepatitis C (HCV) leads to improvements in liver fibrosis. We aimed to investigate the improvement in fibrosis stage following treatment with direct-acting antivirals (DAAs) and factors associated with fibrosis regression. Fibroscan^®^ was performed for patients treated with DAAs, at least 3 years post-HCV eradication. The fibrosis stage at the onset of treatment was compared with the current fibrosis stage. A total of 209 patients were enrolled in this study (56% males; age 58.8 ± 13.3 years; age at treatment 54 ± 10.9 years). Genotype subgrouping was as follows: 1a (16%), 1b (58%), 2a (4%), 3 (18%), and 4a (2%). Overall, 71% of patients were considered treatment-naïve, with a mean follow-up time of 4.5 ± 1.3 years. Fibrosis improvement was observed among 57% of patients; fibrosis progression was seen among 7% of patients and no change was seen in 36% of patients. Moreover, 28% of these patients regressed from F3/F4 to F2 or less. In our multivariable analysis, the age at treatment and advanced fibrosis stage were found to be factors significantly associated with fibrosis regression. In conclusion, fibrosis improvement was observed among 57% of HCV patients after treatment with DAAs. Age and advanced fibrosis at baseline were found to be factors associated with fibrosis regression.

## 1. Introduction

Hepatitis C virus (HCV) is a common cause of chronic hepatitis and a major cause of liver disease; globally, about 58 million people are affected by HCV, with about 1.5 million new cases reported per year. An estimated 290,000 people died from HCV in 2019, mostly from cirrhosis and hepatocellular carcinoma (HCC) [1].

The development of direct-acting antiviral agents (DAAs) and transition to interferon-free oral treatment with high efficacy and a low rate of adverse events revolutionized the treatment of HCV. This was primarily due to the high efficacy rate, simplified treatment regimens, and the ability of general practitioners to prescribe and treat HCV [2]. Curing the HCV infection is the primary goal of current treatment; achieving a sustained virological response (SVR) leads to normalization of the liver enzymes and resolution of the necroinflammatory process and liver fibrosis regression, resulting in improvements in liver function [3,4,5].

The World Health Organization’s (WHO’s) initiative to eliminate HCV was proposed in 2016, targeting a 90% reduction in chronic HCV incidence by the year 2030 and a 65% reduction in mortality compared to the year 2015 [6]. The severity of liver fibrosis is an important predictor of disease progression. The natural history of HCV infection is variable and depends on various factors; hepatic injury can range from minimal necroinflammatory changes to extensive liver fibrosis and cirrhosis development and increases the risk of hepatocellular carcinoma (HCC) development. The updated literature has shown improvements in liver fibrosis (LF) after treatment with DAAs [7,8].

In addition, a decrease in HCC incidence was shown shortly after achieving a SVR in patients with advanced liver fibrosis [7,9,10].

In this present study, we aimed to investigate the predictors of liver fibrosis improvement versus liver fibrosis non-improvement or progression among HCV patients who achieved a SVR with DAAs.

## 2. Materials and Methods

### 2.1. Patients

Patients diagnosed with HCV in the past and achieved a SVR via DAA treatment at least 3 years prior were invited for liver stiffness measurements (LSMs) using vibration-controlled transient elastography (VCTE), Fibroscan^®^ (Echosense, Paris, France). The evaluation included an updated assessment of liver fibrosis severity and stage. The liver fibrosis stage at the treatment time was compared with the updated liver fibrosis stage.

### 2.2. Study Design

A prospective performance of Fibroscan as a modality for liver stiffness was used in addition to retrospective data collection from the time of DAA treatment and onwards. This was a single-center study conducted in the Department of Gastroenterology and Liver Diseases, Soroka University Medical Center (SUMC).

### 2.3. Liver Stiffness Measurement

The updated liver fibrosis assessment was performed using VCTE between 1 February 2022 and 30 September 2022. The updated liver fibrosis stage was compared to the liver fibrosis stage at the time of treatment onset in every patient. The fibrosis stage was established using the following liver stiffness cut-off values: F0/F1 ≤ 7 kPa, F2 > 7 kPa, F3 > 9.6 kPa, and F4 > 14.6 kPa [11]. The following standard criteria for reliable transient elastography were used: 1. at least ten successful measurements; 2. an interquartile range (IQR) lower than 30% of the median value; and 3. a success rate of >60%. The liver stiffness score used was the median of all valid measurements [12]. In addition, the FIB-4 score using age, aspartate aminotransferase (AST), alanine aminotransferase (ALT), and platelets [13], as well as the APRI score using AST, ALT, and platelets were calculated for every patient [14]. The FIB-4 and APRI scores were calculated at the time of treatment and compared to the values calculated during the process of data collection. In addition, a subgroup analysis according to the baseline modality for fibrosis, those who had a Fibroscan^®^ at baseline versus those who had another modality, and the rate of fibrosis regression among both groups were compared.

### 2.4. Data Collection

Clinical data, encompassing data regarding the patient’s demographics, viral information, treatment, and complications, were collected. Comorbidities, such as coinfection with HIV, HBV, HDV, liver steatosis, obesity, diabetes mellitus, hypothyroidism and hyperthyroidism, were collected. Laboratory values were collected at the time of DAA treatment and the most updated values were collected during the prospective assessment. The laboratories included complete blood count (CBC), AST, ALT, alkaline phosphatase, gamma-glutamyl transferase (GGT), total bilirubin, albumin, creatinine, and sodium. Complications related to HCV, including liver cirrhosis, hepatocellular carcinoma, esophageal varices with or without bleeding, and spontaneous bacterial peritonitis (SBP), were also collected in this cohort. In addition, Child–Pugh classification (CPC) was conducted for all cirrhotic patients, allocating 5–6 points for Class A, 7–9 points for Class B, and 10–15 points for Class C.

For every patient, all laboratory values were collected at two time points: shortly before treatment with DAAs and the last available slot during follow-up.

### 2.5. Statistical Analysis

Patient characteristics were presented as mean ± SD for continuous variables and as percentages for categorical variables. Categorical variables were compared using the chi-square test. Continuous variables were examined with the Student’s *t*-test. Continuous variables that were not normally distributed were reported as median (IQR) and compared in the Mann–Whitney U test. Univariate and multivariate analyses were performed. We used logistic regression models to examine the multivariate relationships between the factors underlying liver fibrosis regression after treatment with the DAAs. Before introducing the variables into the model, the multicollinearity of the variables was examined using the variance inflation factor (VIF) statistic. The variables that were found to be significant in the univariate analysis were then introduced into the multivariate model one after the other. All statistical analyses were performed using IBM SPSS version 26 (Chicago, IL, USA). *p*-values less than 0.05 were considered statistically significant. The study protocol was approved by the Institutional Helsinki Committee, approval number 312-21. Informed consent was obtained from all participants.

## 3. Results

### 3.1. Patients

In total, 209 patients were included in this study. The baseline characteristics of this cohort are presented in Table 1. The average age of the patients was 58.02 ± 11.3 years, while the age at treatment time for HCV with the DAAs was 54 ± 10.9 years. Fifty-six percent of the patients were males and 64.6% were immigrants from the former Soviet Union.

### 3.2. Virologic Data

The most common genotype was genotype 1. Thirty-four (16.3%) patients had genotype 1a and 121 patients (57.9%) had genotype 1b, respectively. Thirty-eight (18.2%) patients had genotype 3, while genotypes 2a and 4a had a low rate of prevalence. One hundred and forty-nine (71.3%) patients of this cohort were naïve to HCV treatment. RNA viral loads higher than 800,000 IU/mL were found in 133 (63.6%) patients.

### 3.3. Liver Fibrosis Staging

**Figure 1:**
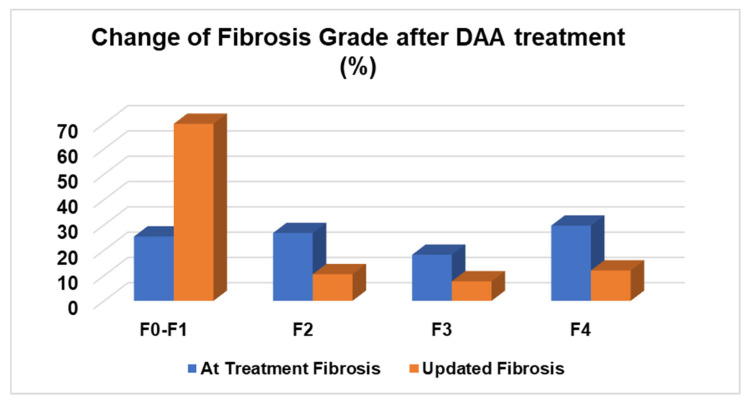
Fibrosis stage during the time of DAA treatment and updated fibrosis stage.

At the time of treatment, liver fibrosis assessments were performed using Fibrotest among 165 (78.9%) patients and Fibroscan among 21 (10%) patients. The assessment during this study was performed using Fibroscan for all enrolled patients. At the time of treatment, the liver fibrosis severity was “F0–F1” in 25.4% of patients, F2 in 26.8% of patients, F3 in 18.2% of patients, and F4 in 29.7% of patients, respectively. In the updated liver fibrosis assessment, the severity was found to be “F0–F1” in 69.9% of patients, F2 in 10.5% of patients, F3 in 7.7% of patients, and F4 in 12% of patients, respectively. Among 35.9% of these patients, no changes in liver fibrosis was observed. A regression of liver fibrosis was observed among 56.9% of patients, while a progression was seen among 7.1% of the cohort. Changes in liver fibrosis staging are presented in Figure 1. A liver fibrosis regression from advanced liver fibrosis of F3/F4 to a lower stage of F2 or less was observed among 58 (27.8%) patients. The different DAAs used in the treatment of these patients are summarized in Table 1. Ninety-nine percent achieved a sustained virologic response (SVR). Two patients who previously did not achieve a SVR after their first treatment were then treated with sofosbuvir/velpatasvir and voxilaprevir and subsequently achieved a SVR.

### 3.4. Comorbidities and Complications

The comorbidities and HCV-related complications are summarized in Table 2. In the context of chronic diagnoses, 23.4% of patients had obesity as a chronic diagnosis, 17.7% had fatty liver disease, and 13.9% had diabetes mellitus. Moreover, 33% (69 patients) of patients had liver cirrhosis, 4.8% had HCC, and 11.5% had esophageal varices. Fifty-eight (84%) of the cirrhotic patients had a Child–Pugh Class A disease and 11 (16%) had Child–Pugh Class B disease at the time of treatment, while sixty-one (88.5%) patients had Class A in the updated data during the study time and eight (11.5%) patients had Class B.

### 3.5. Laboratory Values

The different laboratory variables at the time of treatment and updated values during the study are presented in Table 3. Significant improvements in the liver enzymes at the study time compared to the treatment time were observed. The mean ALT significantly decreased from 64.9 ± 47 at the time of treatment to 20.5 ± 12.2 at time of our study (*p* < 0.001). AST decreased from 60.6 ± 39 to 29 ± 29 (*p* < 0.001), GGT from 74 ± 71.4 to 35.6 ± 36.1 (*p* < 0.001), and alkaline phosphatase from 91.1 ± 31.7 to 79.3 ± 43.3 (*p* < 0.001) during this time frame. Significant improvement in the APRI score was observed after HCV treatment, with an average decrease from 1.4 ± 1.7 to 0.93 ± 2.4 (*p* = 0.028). There was also a non-significant increase in the FIB-4 score after the treatment despite the decrease in ALT and AST, from 3.09 ± 4 to 3.5 ± 8.8 (*p* = 0.505).

### 3.6. Liver Fibrosis Regression

A comparison of patients with liver fibrosis regression and those without liver fibrosis regression is presented in Table 4. Significant differences were found between these two groups with regard to several variables. Patients with regression in liver fibrosis were older, diagnosed at an older age, and treated at an older age, 59.4 ± 10.7 vs. 56.1 ± 11.9, *p* = 0.018, 48.6 ± 11.8 vs. 44.9 ± 12, *p* = 0.027, and 55.5 ± 10.5 vs. 52.1 ± 11.4, *p* = 0.029, respectively. A higher rate of males was found among the liver fibrosis regression group: 75 patients (63%) compared to 42 patients (46.7%), *p* = 0.018.

Significant differences were observed in liver fibrosis staging at the baseline DAA treatment time and at the time of study performance. Among the patients with fibrosis regression, higher rates of advanced fibrosis were found at the baseline (F2 37.8%, F3 27.7%, and F4 33.6%, respectively) compared to the group with fibrosis non-regression, in which higher rates of mild/early fibrosis were observed (“F0–F1” 57.8%, F2 12.2%, F3 5.6%, and F4 22.4%, respectively), *p* < 0.001. Regarding the updated liver fibrosis staging, significantly higher rates of mild liver fibrosis stages were observed among the groups with liver fibrosis regression compared to the non-regression group (“F0–F1” 78% vs. 59%, F2 12.6% vs. 4.4%, F3 9.25% vs. 5.6%, and F4 0 vs. 27.8%, respectively, *p* < 0.001).

Focusing on the liver-related complications, no significant differences were found regarding the liver cirrhosis rate, HCC, SBP, or Child–Pugh classification between the two groups, but significantly lower rates of esophageal varices and esophageal varices bleeding were found among the liver fibrosis regression groups (7.6% vs. 16.7%, *p* = 0.043, and 1.7% vs. 10%, *p* = 0.008, respectively).

No significant differences were found regarding the genotypes, rates of experienced patients, or rates of patients with a viral load higher than 800,000 IU/mL.

No significant differences were found regarding the comorbidities between the groups (HIV, HBV, HDV, DM, fatty liver disease, obesity, non-Hodgkin lymphoma, depression, hypothyroidism, or hyperthyroidism).

The laboratory results of the different groups are presented in Tables S1 and S2. The baseline values at the time of treatment compared between the patients with regressed liver fibrosis and those without regressed liver fibrosis can be found in Table S1. The updated laboratory values compared in each study group can be seen in Table S2. No significant difference was found between the groups for most laboratory values at the time of treatment; however, significant improvements in several values were found in both groups regarding the updated values compared to the values before DAA treatment (ALT, AST, and GGT).

The univariate and multivariate analyses for factors associated with regression in liver cirrhosis are presented in Table 5. In the univariate analysis, the age, age at treatment, age at diagnosis, male gender, and the fibrosis stage (OR 1.027, 95% CI 1.002–1.052, *p* = 0.038, OR 1.029, 95% CI 1.003–1.055, *p* = 0.031, OR 1.026, 95% CI 1.003–1.051, *p* = 0.029, and OR 2.209, 95% CI 1.716–2.845, *p* < 0.001, respectively) were significant factors associated with liver fibrosis regression. There was no significant association between liver fibrosis regression and viral load above 800,000 IU/mL, or liver fibrosis regression and the level of ALT, AST, or platelets at the time of treatment. However, in the multivariate analysis, only the age at treatment and fibrosis stage were found to be factors significantly associated with fibrosis regression after treatment with DAAs (OR 1.359, 95% CI 1.055–1.751, *p* = 0.017 and OR 2.555, 95% CI 1.864–3.503, *p* < 0.001, respectively).

With regard to the FIB-4 and APRI scores, we found a fibrosis regression rate of 49% in the patients according to FIB-4, and 59% according to the APRI score, respectively.

In a subgroup analysis of our cohort between those who had a Fibroscan at baseline versus those who underwent another modality, 10% (21 patients) of patients in our cohort had Fibroscan^®^ at baseline, and in a comparison of the results of the Fibroscan^®^ at baseline to the updated Fibroscan^®^, we found 48% of patients experienced fibrosis regression, while a regression rate of 59% was found in all other patients (*p* = 0.338).

## 4. Discussion

Our study demonstrated liver fibrosis regression among 57% of HCV patients treated with DAAs. Overall, 35% of these patients were found to have a stable fibrosis stage, while fibrosis progression was found among 5% of these patients. In addition, about 28% of these patients had fibrosis regression from advanced fibrosis stage 3 or 4 to stage 2 or less. Multivariate analysis revealed age and fibrosis severity as factors significantly associated with liver fibrosis regression after treatment with DAAs. Due to the high cure rate of HCV following treatment with DAAs, there has a been a shift in interest from success rates to long-term outcomes of treatment with DAAs and several different other unmet needs of HCV patients, such as cirrhosis decompensation or regression, HCC frequency, and mortality rates [15]. The WHO has begun an initiative to eliminate HCV [16], targeting a 90% reduction in chronic HCV incidence and a 65% reduction in HCV mortality by the year 2030 [6].

Liver fibrosis regression is an important variable to study as it has been shown to decrease complications, such as HCC, and the need for transplantation. Several studies reported a reduction in the rate of HCC following DAA treatment as a result of liver fibrosis regression [7,9,16,17,18].

Different rates of fibrosis regression have been reported in previous studies. In comparing our results with those in the literature, we found an improvement of liver fibrosis in the majority of patients (60% of patients). One previous study demonstrated a rate of fibrosis regression of 42% [19]. In another study of patients with a fibrosis stage of F3 (using transient elastography values between 9.6 kPa and 14.6 kPa), fibrosis regression was seen in 58% of patients, stable fibrosis was seen in 30% of patients, and fibrosis progression was seen in 12.5% of patients treated with DAAs [7]. Studies have shown mild fibrosis regression shortly after treatment and delayed regression in patients with advanced fibrosis at baseline [8]. A study that included 95 patients reported a decrease in liver stiffness among patients treated with DAAs: 29% after 48 weeks and 39% at 144 weeks, respectively [20]. According to these studies and other studies published in recent years, it is obvious that liver fibrosis regression after treatment with DAAs occurs in a large number of patients; however, it is still unclear why some patients remain stable and progress. Long-term outcomes of patients after liver fibrosis regression also remains unknown. Possible causes of fibrosis progression and non-improvement may be related to advanced liver fibrosis/cirrhosis at the beginning of the treatment. Advanced stage results in longer time before liver tissue healing and may prevent healing altogether; other causes could be related to liver-related comorbidities, particularly if the patients had a combination of liver diseases, such as hepatitis C in combination with hepatitis B infection or fatty liver disease. In addition, other non-liver comorbidities, such as diabetes mellitus, obesity, and metabolic syndrome, could increase the risk for liver fibrosis and could result in the progression of liver fibrosis unrelated to liver disease [21,22].

In our study, we performed multivariate analysis in order to identify the specific factors associated with liver fibrosis regression. The age at treatment and advanced fibrosis at the treatment time were found to be factors associated with an increased regression rate; however other factors, such as gender, viral load, ALT, AST, and platelets, were not found to be associated with fibrosis regression.

There are several non-consistent findings in previous studies regarding possible factors that affect liver fibrosis regression. Specific factors, such as a sustained viral response, age <40 years, body mass index <27, no or minimal baseline activity, and a viral load <3.5 millions copies per milliliter, have been found to be associated with fibrosis regression [23]; however, parts of these factors, such as SVR and viral load, are no longer relevant due to the high efficacy of the DAAs.

In patients treated with DAAs, the absence of diabetes mellitus type 2 and the presence of a high platelet count were independently associated with improvements in liver stiffness [19]. Another study that investigated fibrosis regression 12 months post-treatment found that elevated baseline ALT and genotype 1 predicts the fibrosis regression after treatment with DAAs [24]. Furthermore, a reduction of 2.1 kPa was reported at the time of SVR12, with a higher fibrosis regression rate among patients with advanced fibrosis compared to those with null-mild fibrosis [25]. Taken together, it seems that the advanced fibrosis stage at the baseline time and the age/duration of the disease predicts the fibrosis regression; however, no clear sufficient data exist with regard to other predictors, such as ALT/AST, SVR, and others. Additional studies with large numbers of patients are needed for investigating specific factors that are associated with fibrosis regression, such as genotype, baseline labs, liver-related and non-liver related comorbidities, and ethnicity.

To summarize, a longitudinal improvement in liver fibrosis was found following the treatment of HCV with DAAs, resulting in a decrease in complications and mortality [26,27], and even an improvement of non-liver complications, such as diabetes mellitus, chronic kidney disease, cardiovascular disease, and non-liver cancer [28]. Additional positive effects on other organs were reported, such as brain volume changes, and improvements in different functions were also outlined, such as attention, planning, working, and memory, along with further improvements in fatigue and depression [29,30]. Nowadays, there is no doubt around the importance of hepatitis C treatment; however, several important points need to be highlighted. First, many barriers must be overcome in order to increase the screening and treatment rates of HCV and reaching the goals as suggested in the WHO initiative [31,32]. Second, long-term complications of patients with liver fibrosis regression from advanced to mild need to be carefully monitored and followed up.

The strengths of the present study lie in a prospective fibrosis assessment using Fibroscan and the large number of patients included. However, there are several limitations to mention: the study includes several different DAAs since the year 2015, some of which are no longer used in the era of pan genotypic DAAs. Another limitation of the study is that most of our patients had HCV genotype 1, and this study was performed in a single-center setting. Lastly, bias of the selected patients could affect the study results, as it is possible that only motivated patients agreed and adhered to participation in the study. Another important limitation is that the prospective part of this study used Fibroscan for fibrosis assessment while most patients performed Fibrotest at the baseline fibrosis assessment. However, we used FIB-4 and APRI scores for additional fibrosis assessments and performed a subgroup analysis according to the baseline modality used and found a fibrosis regression range between 48% and 59%, which supports our findings.

## 5. Conclusions

Liver fibrosis regression was observed in most HCV patients treated with DAAs. Age at treatment and fibrosis stage were found to be factors significantly associated with fibrosis regression. Patients with HCV should be encouraged for treatment with DAA treatment.

## Figures and Tables

**Table 1:** Baseline characteristics of the study cohort.

Characteristic	Patients, n = 209 (%)
Age, mean ± SD, years	58 ± 11.3
Age at diagnosis, mean ± SD, years	47 ± 12.1
Age at treatment, mean ± SD, years	54 ± 10.9
Sex (male)	117 (56)
Ethnicity	
Jewish	201 (96.2)
Bedouin	8 (3.8)
Subgrouping	
Israeli-born	74 (35.4)
Immigrant—former Soviet Union	135 (64.6)
Genotype	
1a	34 (16.3)
1b	121 (57.9)
2a	9 (4.3)
3	38 (18.2)
4a	4 (1.9)
Patient type	
Treatment-naïve	149 (71.3)
Experienced past treatment	58 (27.8)
Viral load (IU/mL)	
≤800,000	76 (36.4)
>800,000	133 (63.6)
Method of fibrosis assessment at treatment time	
Fibrotest	165 (78.9)
Fibroscan	21 (10)
Fibrosis stage at treatment time	
F0–F1	53 (25.4)
F2	56 (26.8)
F3	38 (18.2)
F4	62 (29.7)
Updated fibrosis stage	
F0–F1	146 (69.9)
F2	22 (10.5)
F3	16 (7.7)
F4	25 (12)
Change in fibrosis score	
No change in the fibrosis score	75 (35.9)
Regression of fibrosis	
3 stages	18 (8.6)
2 stages	38 (18.2)
1 stage	63 (30.1)
Progression of fibrosis	
1 stage	12 (5.7)
2 stages	3 (1.4)
Regression in the fibrosis score	
From F3/F4 to F2 or less	58 (27.8)
Treatment type	
Dasabuvir/ombitasvir or paritaprevir/ritonavir	19 (9.1)
Dasabuvir/ombitasvir or paritaprevir/ritonavir/ribavirin	13 (6.2)
Ledipasvir/sofosbuvir	28 (13.4)
Ledipasvir/sofosbuvir/ribavirin	4 (1.9)
Elbasvir/grazoprevir	39 (18.7)
Daclatasvir/sofosbuvir	4 (1.9)
Daclatasvir/sofosbuvir/ribavirin	16 (2.9)
Sofosbuvir/velpatasvir	49 (23.4)
Pibrentasvir/glecaprevir	45 (21.5)
Sustained virologic response (SVR)	207 (99)

**Table 2:** Comorbidities of the study cohort.

Comorbidity	Patients, n = 209 (%)
HIV	5 (2.4)
HBV	7 (3.3)
HBV/HDV	1 (0.5)
Fatty liver disease	37 (17.7)
Obesity	49 (23.4)
Diabetes mellitus	29 (13.9)
Non-Hodgkin lymphoma	3 (1.4)
Depression	16 (7.7)
Hypothyroidism	14 (6.7)
Hyperthyroidism	2 (1)
Complication	
Liver cirrhosis	69 (33)
HCC	10 (4.8)
Esophageal varices	24 (11.5)
Esophageal varices bleeding	11 (5.3)
SBP	2 (1)
Child–Pugh classification—at treatment time (n = 69)	
A	58 (84)
B	11 (16)
C	0
Child–Pugh classification—updated (n = 69)	
A	61 (88.5)
B	8 (11.5)
C	0

**Table 3:** Laboratory values at the treatment time and updated values.

Laboratory	At Treatment	Updated Value	*p*-Value
Hemoglobin	13.86 ± 1.7	13.43 ± 1.69	<0.001
WBC	6.9 ± 2.2	8.3 ± 12.9	0.107
PLT	212 ± 232	210 ± 84	0.876
INR	1.00 ± 0.14	1.02 ± 0.16	0.022
ALT	64.9 ± 47	20.5 ± 12.2	<0.001
AST	60.6 ± 39	29 ± 29	<0.001
GGT	74 ± 71.4	35.6 ± 36.1	<0.001
Alkaline phosphatase	91.1 ± 31.7	79.3 ± 43.3	<0.001
Total bilirubin	0.79 ± 0.5	0.0.71 ± 0.63	0.077
Creatinine	0.69 ± 0.17	0.77 ± 0.38	<0.001
Albumin (gr/dL)	4.10 ± 0.41	4.12 ± 0.4	0.519
Sodium	138.5 ± 13	139.0 ± 16	0.676
Fib-4 score	3.09 ± 4	3.5 ± 8.8	0.505
APRI score	1.4 ± 1.7	0.93 ± 2.4	0.028

**Table 4:** Characteristics of patients with fibrosis regression compared to the non-regression or progression group.

Characteristic	Fibrosis Regression n = 119 (%)	No Fibrosis Regression n = 90 (%)	*p*-Value
Age	59.4 ± 10.7	56.1 ± 11.9	0.018
Age at diagnosis	48.6 ± 11.8	44.9 ± 12	0.027
Age at treatment	55.5 ± 10.5	52.1 ± 11.4	0.029
Sex (male)	75 (63)	42 (46.7)	0.018
Ethnicity			
Jewish	84 (93.3)	117 (98.3)	
Bedouin	2 (1.7)	6 (6.7)	0.130
Genotype			0.664
1a	16 (17.8)	18 (15.1)	
1b	54 (60)	67 (56.3)	
2a	5 (5.6)	4 (3.4)	
3	13 (14.4)	25 (21)	
4a	2 (2.2)	2 (1.6)	
Patient type			0.984
Naïve	85 (72)	64 (71.9)	
Experienced	33 (28)	25 (28.1)	
Viral load (IU/mL)			0.833
≤800,000	44 (37)	32 (35.6)	
>800,000	75 (63)	58 (64.4)	
Method of fibrosis assessment at treatment time			0.052
Fibrotest	101 (78.4)	64 (77.1)	
Fibroscan	10 (8.8)	11 (13.3)	
Fibrosis stage at treatment time			<0.001
F0–F1	1 (0.8)	52 (57.8)	
F2	45 (37.8)	11 (12.2)	
F3	33 (27.7)	5 (5.6)	
F4	40 (33.6)	22 (22.4)	
Updated fibrosis stage			<0.001
F0–F1	93 (78)	53 (59)	
F2	15 (12.6)	4 (4.4)	
F3	11 (9.25)	5 (5.6)	
F4	0	25 (27.8)	
Sustained virologic response (SVR)	117 (98.3)	90 (100)	0.217
Liver cirrhosis	42 (36.2)	27 (30.7)	0.409
HCC	4 (3.5)	6 (6.9)	0.267
Esophageal varices	9 (7.6)	15 (16.7)	0.043
Esophageal varices bleeding	2 (1.7)	9 (10)	0.008
SBP	0	2 (2.2)	0.104
Child–Pugh classification—at treatment time			0.650
A	36 (85.7)	22 (81.5)	
B	6 (84.3)	5 (18.5)	
C	0		
Child–Pugh classification—updated			0.719
A	37 (85.7)	24 (88.9)	
B	5 (14.3)	3 (11.1)	
C	0	0	

**Table 5:** Univariate and multivariate analyses of the fibrosis regression factors.

	Univariate Analysis			Multivariate Analysis		
	OR	95% CI	*p*-Value	OR	95% CI	*p*-Value
Age	1.027	1.001–1.052	0.038	0.713	0.552–0.919	0.009
Age at treatment	1.029	1.003–1.055	0.031	1.359	1.055–1.751	0.017
Age at diagnosis	1.026	1.003–1.051	0.029	1.039	0.986–1.094	0.155
Sex, male	1.948	1.116–3.399	0.019	1.151	0.594–2.231	0.677
Baseline fibrosis stage	2.209	1.716–2.845	<0.001	2.555	1.864–3.503	<0.001
Viral load > 800,000 IU/mL	0.940	0.532–1.663	0.833			
ALT at treatment	1.003	0.997–1.009	0.288			
AST at treatment	1.003	0.996–1.011	0.382			
Platelets at treatment	0.999	0.998–1.001	0.393			

## Data Availability

No additional data are available.

## Supplementary Materials

The following supporting information can be downloaded at: https://www.mdpi.com/article/10.3390/life13091872/s1, Table S1: Laboratory values of fibrosis regression and non-regression groups; Table S2: Laboratory values at the time of treatment and updated values in the subgroups.
