# Supplementary material for: Liver Fibrosis Regression and Associated Factors in HCV Patients Treated with Direct-Acting Antiviral Agents
Source: Life (Basel). 2023 Sep 5;13(9):1872. doi: 10.3390/life13091872 (PMC10533124; doi:10.3390/life13091872)
Supplement: Supplementary file 1 [file life-13-01872-s001.zip › life-2598295-supplementary.pdf]

Table S1: laboratory values of patients with fibrosis regression compared non regression group

Laboratories at treatment time	Regression	No regression or progression	P-value
Hemoglobin	13.85±1.6	13.88±1.8	0.889
WBC	6.7±1.9	7.1±2.7	0.177
PLT	199±83	231±341	0.321
ALT	57.8±36	60.9±40	0.271
AST	62.7±41	57.9±36	0.383
GGT	82.6±82	63±51	0.021
Alkaline Phosphatase	89.8±32	92.8±31	0.255
Albumin	4.1±0.4	4.0±0.4	0.347
Fib-4 score	3.0±4	3.2±4	0.982
APRI score	1.39±1.78	1.4±1.77	0.752

Table S2: laboratory values at the time of treatment and updated values in the subgroups

Patients with regressed fibrosis, n=119			
Laboratories	At treatment time	Updated values	p-value
Hemoglobin	13.8±1.65	13.4±1.7	<0.001
WBC	6.7±1.9	7.4±2.4	<0.001
PLT	199±83	219±81	<0.001
ALT	67.9±51	20.0±13	<0.001
AST	62.7±41	29.6±31	<0.001
GGT	82.3±82	33.6±35	<0.001
Alkaline Phosphatase	89.8±32	77.8±46	0.008
Albumin	4.1±0.38	4.15±0.43	0.362
Fib-4 score	3.0±4	2.6±4.3	0.404
APRI score	1.4±1.8	0.69±1.4	<0.001
Patients without fibrosis regression, n=90			
Hemoglobin	13.9±1.8	13.4±1.6	<0.001
WBC	7.16±2.7	9.5±19.5	0.243
PLT	231±341	198±86	0.344
ALT	60.9±40	21.3±10.9	<0.001
AST	57.9±35	27.8±21	<0.001
GGT	63±51	38.4±37	<0.001
Alkaline Phosphatase	92.8±31	81.4±38	0.005
Albumin	4.0±0.4	4.0±0.35	0.884
Fib-4 score	3.19±4	4.83±12.7	0.257
APRI score	1.4±1.7	1.27±3.4	0.750
